# Intravesical hyaluronic acid and chondroitin sulphate for recurrent urinary tract infections: a prospective multicentre comparison of two instillation protocols

**DOI:** 10.3389/fsurg.2026.1897487

**Published:** 2026-09-07

**Authors:** Marilena Gubbiotti, Alessandro Giammò, Enrico Ammirati, Annarita Cicalese, Elisabetta Costantini, Ester Illiano, Vincenzo Li Marzi, Mara Bacchiani, Leonardo Martino, Vito Mancini, Stefano Rosadi

**Affiliations:** 1 Department of Urology, Valdarno Hospital, USL Toscana Sud-Est, Montevarchi, Italy; 2 Department of Neuro-Urology, City of Health and Science University Hospital, Turin, Italy; 3 Department of Urology, San Giuseppe Moscati Hospital, Avellino, Italy; 4 Andrology and Urogynecology Clinic, Santa Maria Hospital, Terni, Italy; 5 Department of Medical, Surgical and Neurosciences, University of Siena, Siena, Italy; 6 Unit of Oncologic Minimally-Invasive Urology and Andrology, Careggi Hospital, Firenze, Italy; 7 Department of Urology and Renal Transplantation, University of Foggia, Foggia, Italy; 8 Department of Urology, Molfetta Hospital, Bari, Italy

**Keywords:** chondroitin sulphate, glycosaminoglycan, hyaluronic acid, intravesical therapy, lower urinary tract symptoms, non-antibiotic treatment, recurrent urinary tract infections

## Abstract

**Background:**

Recurrent urinary tract infections (r-UTIs) are highly prevalent in women and are associated with reduced quality of life and increased healthcare burden. Intravesical glycosaminoglycan (GAG) replenishment with hyaluronic acid (HA) and chondroitin sulphate (CS) has shown clinical benefit, but the optimal instillation schedule remains undefined. This study aimed to compare the safety and effectiveness of two HA + CS instillation protocols in women with r-UTIs.

**Methods:**

In this prospective multicentre observational study, women ≥18 years with r-UTIs, defined according to European Association of Urology guidelines, were enrolled. Patients received HA + CS according to institutional practice: Group A (weekly instillations for 4 weeks followed by monthly maintenance for 12 months) or Group B (weekly instillations for 8 weeks followed by monthly maintenance for 12 months). Clinical evaluation, 3-day bladder diary, uroflowmetry (UF), post-void residual (PVR), visual analogue scale (VAS) for pelvic pain, urinalysis, urine culture, and ICIQ-FLUTS questionnaire were assessed at baseline and during follow-up (1, 3, 6, and 12 months). The primary outcome was the cumulative number of UTI episodes recorded throughout the entire 12-month follow-up period.

**Results:**

A total of 140 women (70 per group) were included. At 12 months, mean UTI episodes decreased to 0.6 ± 0.5 in Group A and 0.7 ± 0.3 in Group B, without significant between-group differences (*p* > 0.05). Storage and voiding symptoms, pelvic pain (VAS), and ICIQ-FLUTS scores significantly improved from baseline in both groups (all *p* < 0.001). UF parameters and PVR remained stable. No serious adverse events were observed.

**Conclusion:**

Intravesical HA + CS significantly reduced UTI recurrence and improved urinary symptoms over 12 months. No statistically significant differences in clinical outcomes were observed between the two treatment groups over the 12-month follow-up.

## Introduction

Recurrent urinary tract infections (r-UTIs) represent a frequent and challenging condition in urological practice, particularly affecting adult and postmenopausal women, with a substantial impact on quality of life (QoL) and healthcare resource utilization. According to the European Association of Urology (EAU) guidelines, r-UTIs are defined as at least two symptomatic episodes within six months or three episodes within one year, confirmed by positive urine cultures (1). Despite their high prevalence, the optimal long-term management of r-UTIs remains controversial (2).

For decades, antibiotic prophylaxis has been the cornerstone of r-UTI prevention. Although effective in reducing recurrence rates, prolonged or repeated antibiotic exposure is associated with increasing antimicrobial resistance, adverse drug reactions, and alterations of the urinary and gut microbiota (3, 4). These limitations have encouraged the development of non-antibiotic preventive strategies aimed at addressing the underlying pathophysiological mechanisms involved in r-UTIs.

Among these mechanisms, impairment of the bladder urothelial barrier, particularly the glycosaminoglycan (GAG) layer, plays a central role in bacterial adhesion, urothelial inflammation, and increased susceptibility to infection (5). The GAG layer is a hydrophilic anti-adherence barrier that limits bacterial binding to the urothelium and regulates bladder permeability. Disruption or depletion of this layer has been demonstrated in patients with r-UTIs and other chronic inflammatory bladder conditions, contributing to symptom persistence and recurrence (5). Intravesical therapies aimed at restoring the GAG layer have therefore gained increasing attention. Hyaluronic acid (HA) and chondroitin sulphate (CS), two essential components of the urothelial extracellular matrix, exert barrier-restoring, anti-inflammatory, and anti-adherence effects. Several clinical studies have shown that intravesical instillations of HA and CS are safe and effective in reducing UTI recurrence rates, improving storage lower urinary tract symptoms (LUTS), and alleviating pelvic pain (6–8). Despite the growing body of evidence supporting GAG replenishment therapy, there is currently no standardized protocol regarding the optimal frequency and duration of intravesical HA + CS instillations. In daily clinical practice, heterogeneous regimens are adopted, ranging from short induction cycles to more intensive protocols with prolonged maintenance phases, often based on empirical experience rather than comparative evidence (6, 7). This lack of standardization represents a significant gap in the literature, with important implications for patient adherence, treatment costs, and healthcare resource allocation. The aim of this prospective multicentre study was to compare the safety and efficacy of two commonly adopted intravesical HA + CS instillation protocols in women with r-UTIs over a 12-month follow-up period.

## Materials and methods

This was a prospective, multicentre pragmatic comparative cohort study conducted in six Italian referral centres with expertise in female LUTS management. The study was designed to evaluate the clinical outcomes of two different intravesical instillation protocols using HA and CS in women with r-UTIs.

Women aged 18 years or older with a diagnosis of r-UTIs were consecutively enrolled. The study was conducted in accordance with the Declaration of Helsinki and was approved by the Institutional Review Board of Valdarno Hospital. Written informed consent was obtained from all participants prior to enrolment. R-UTIs were defined in accordance with the EAU guidelines on urological infections as recurrent symptomatic episodes confirmed by positive urine culture (1). All participants provided a documented history of recurrent infections prior to enrolment.

Exclusion criteria included age <18 years, neurogenic bladder, the presence of pelvic organ prolapse ≥ II according to the Pelvic Organ Prolapse Quantification (POP-Q) system, significant PVR (>50 mL), urolithiasis, interstitial cystitis (IC), history of bladder malignancy or pelvic radiotherapy, complicated UTIs, renal insufficiency, diabetes mellitus, current corticosteroid use, pregnancy, uncontrolled systemic diseases, immunosuppressive therapy, ongoing antibiotic prophylaxis.

At baseline, all patients underwent a comprehensive clinical evaluation, including detailed medical and urological history, urogynecological examination, 3-day bladder diary, uroflowmetry (UF), post-void residual (PVR) volume evaluation, urinalysis, and urine culture. Pelvic pain was assessed using a visual analogue scale (VAS; 0 = worst; 10 = best). No participants were receiving antibiotic therapy when intravesical HA + CS treatment was started.

### Treatment protocols

Intravesical treatment (Ialuril® Prefill, 50 mL: HA 1.6%, CS 2%, IBSA Farmaceutici Italia) was administered according to two different protocols. Treatment allocation was not randomized but reflected routine clinical practice at each participating centre, consistent with the pragmatic design of the study. Each participating centre routinely adopted one predefined instillation protocol throughout the study period. Consequently, all consecutive eligible patients treated at a given centre received the same protocol according to local clinical practice, without investigator- or patient-driven treatment selection. Patients assigned to Group A received intravesical instillations of HA + CS once weekly for four consecutive weeks (induction phase), followed by a maintenance phase consisting of monthly instillations for 12 months. Patients assigned to Group B received intravesical instillations of HA + CS once weekly for eight consecutive weeks (induction phase), followed by monthly maintenance instillations for 12 months.

All instillations were performed by trained healthcare professionals using standardized aseptic techniques. Patients were instructed to retain the instilled solution for the maximum tolerable time, according to routine clinical practice.

All previous preventive therapies (including antibiotics, urinary antiseptics, cranberry, D-mannose, probiotics, vaginal estrogen therapy, immunomodulatory agents, and other supportive treatments) were discontinued, and no concomitant preventive therapies were administered during the study.

### Follow-up and outcome assessment

Follow-up evaluations were scheduled at 1, 3, 6, and 12 months after treatment initiation. At each follow-up visit, patients underwent clinical reassessment including symptom evaluation through a 3-day bladder diary, VAS score for pelvic pain, UF, PVR urine volume assessment, urinalysis, and urine culture. Patients were also systematically evaluated for the occurrence of adverse events related to intravesical instillations. In addition, patients completed the International Consultation on Incontinence Questionnaire-Female Lower Urinary Tract Symptoms (ICIQ-FLUTS) at baseline and at the final follow-up visit to assess changes in patient-reported outcomes over time. The ICIQ-FLUTS is a validated, self-administered questionnaire specifically designed to evaluate the presence and severity of female LUTS, including storage, voiding, and incontinence domains, as well as their impact on quality of life (QoL), with higher scores indicating greater symptom severity. The questionnaire has been extensively validated and is widely used in both clinical practice and research settings; a validated Italian version was available and used in the present study (9). The questionnaire was administered at baseline and at the final follow-up visit. Previous pharmacological treatments were evaluated. The primary outcome was the cumulative number of recurrent UTI episodes recorded over the entire 12-month follow-up period. Secondary outcomes included changes in storage LUTS, pelvic pain intensity, and UF parameters. UTI recurrence was defined as the presence of urinary symptoms associated with a positive urine culture.

#### Statistical analysis

Descriptive statistics were used to summarize baseline characteristics and clinical outcomes. Continuous variables were expressed as mean ± standard deviation (SD) as appropriate, while categorical variables were reported as counts and percentages. The distribution of continuous variables was assessed using the Shapiro–Wilk test. Between-group comparisons (Group A vs. Group B) were performed using Student's t-test for normally distributed variables and the Mann–Whitney U test for non-normally distributed variables. Additional exploratory sensitivity analyses were performed to assess the potential influence of participating centres on the primary outcome. Categorical variables were compared using the chi-square test or Fisher's exact test, as appropriate. Within-group changes from baseline to follow-up were evaluated using paired t-tests or Wilcoxon signed-rank tests according to data distribution. For outcomes assessed at multiple follow-up timepoints (1, 3, 6, and 12 months), comparisons were conducted between each follow-up visit and baseline, as well as between groups at corresponding timepoints. No adjustment for multiple comparison was performed due to the exploratory nature of secondary endpoints. The primary outcome (number of UTI episodes during the follow-up period) was analyzed as a continuous variable and compared between groups according to data distribution. Secondary outcomes included changes in storage symptoms, pelvic pain (VAS), uroflowmetry parameters (Qmax), post-void residual volume (PVR), and patient-reported outcomes (ICIQ-FLUTS), which were analyzed using the same statistical approach described above.

All statistical tests were two-sided, and a *p*-value < 0.05 was considered statistically significant. Statistical analyses were performed using IBM SPSS Statistics for Windows, Version 27.0 (IBM Corp., Armonk, NY, USA). A sample size of 70 patients per group, assuming a two-sided significance level (*α*) of 0.05 and a statistical power of 80%, was considered sufficient to detect a standardized between-group difference (Cohen's d) of approximately 0.48, corresponding to a moderate effect size for comparisons between the two treatment groups.

## Results

A total of 140 women with r-UTIs were prospectively enrolled across six Italian referral centres. Seventy patients were allocated to Group A (4-week induction + monthly maintenance), and seventy to Group B (8-week induction + monthly maintenance). Mean age (±SD) was 57.2 ± 17.7 years in Group A and 56.0 ± 14.3 years in Group B, with no significant difference between groups. No patients were receiving antibiotic therapy at the initiation of HA + CS treatment.

Baseline comorbidities were similarly distributed between groups (*p* = 0.70; Table 1). The mean duration of r-UTIs history was 7.2 ± 8.4 years in Group A and 6.6 ± 8.4 years in Group B (*p* = 0.60). The mean number of UTI episodes in the 12 months prior to enrolment was 6.5 ± 3.4 and 7.6 ± 5.7 in Group A and B, respectively, without statistically significant differences (*p* = 0.80). The mean number of previous pharmacological treatment attempts was 4.4 ± 3.2 in Group A and 4.8 ± 4.1 in Group B (*p* = 0.76). Previous treatments included multiple pharmacological and non-pharmacological approaches, such as repeated antibiotics courses, urinary antiseptics, phytotherapeutic agents (including cranberry and D-mannose), probiotics, vaginal estrogen therapy, immunomodulatory therapies, and other supportive treatments, often in combination. All enrolled patients completed the scheduled intravesical instillation protocol according to their assigned treatment regimen, with no treatment discontinuations or dropouts during the study.

**Table 1:** Distribution of baseline comorbidities in the overall study population and in groups A and B.

Comorbidity	Overall (*n* = 140)	Group A (*n* = 70)	Group B (*n* = 70)
Comorbidity ≥1, *n* (%)	104 (*74.3%)*	48 (*68.6%)*	56 (*80%)*
Cardiovascular	22 (*15.7%)*	9 (*12.8%)*	13 (*18.6%)*
Endocrine	13 (*9.3%)*	5 (*7.1%)*	8 (*11.4%)*
Autoimmune	31 (*22.1%)*	12 (*17.1%)*	19 (*27.1%)*
Gastrointestinal	20 (*14.3%)*	9 (*12.8%)*	11 (*15.7%)*
Neurological	10 (*7.1%)*	4 (*5.7%)*	6 (*8.6%)*
Gynecological	11 (*7.9%)*	4 (*5.7%)*	7 (*10.0%)*
Other	38 (*27.1%)*	18 (*25.7%)*	20 (*28.6%)*

### Primary outcome: UTI recurrence

Both treatment protocols were associated with a marked reduction in UTI recurrence over the 12-month follow-up period. At 12 months, the mean number of UTI episodes decreased to 0.6 ± 0.5 in Group A and 0.7 ± 0.3 in Group B (*p* > 0.05). No statistically significant difference was observed between the two instillation protocols at the final follow-up (*p* > 0.05). Exploratory sensitivity analyses accounting for participating-centre variability did not substantially modify the observed between group results.

### Functional outcomes

At baseline, storage symptoms were highly prevalent in the overall cohort, with increased daytime frequency reported by more than 75% of patients, nocturia by 68%, and urgency by over 90%, without significant differences between treatment groups (Table 2). A progressive and clinically meaningful improvement in storage symptoms was observed in both groups starting from the first follow-up visit and maintained throughout the 12-month observation period. At the final follow-up, only a minority of patients continued to report storage symptoms. No statistically significant differences were detected between Group A and Group B at any timepoint (*p* > 0.05). Urinary incontinence episodes showed a similar decreasing trend over time in both treatment arms (*p* = 0.8). Pelvic pain, evaluated using the VAS, showed consistent improvement during follow-up in both groups, with progressive changes maintained up to 12 months. Between-group comparisons did not demonstrate statistically significant differences (all *p* > 0.05).

**Table 2:** Urinary symptoms, VAS score, and uroflowmetry parameters, at baseline and during follow- up in groups A and B.

	Pre-instillations		1-month follow-up		3-month follow-up		6-month follow-up		12-month follow-up	
	Group A	Group B	Group A	Group B	Group A	Group B	Group A	Group B	Group A	Group B
Increased day- time urinary frequency *n* (%)	56/70 (80%)	53/70 (75.7%)	48/70 (68.6%)	44/70 (62.8%)	23/70 (32.8%)	20/70 (28.6%)	9/70 (12.8%)	7/70 (10%)	8/70 (11.4%)	7/70 (10%)
Increased night- time urinary frequency *n* (%)	51/70 (72.8%)	46/70 (65.7%)	39/67 (55.7%)	37/70 (52.8%)	18/70 (25.7%)	13/70 (18.6%)	7/70 (10%)	8/70 (11.4%)	6/70 (8.6%)	5/70 (7.1%)
Urgency episodes/day *n* (%)	63/70 (90%)	67/70 (95.7%)	43/70 (61.4%)	46/70 (65.7%)	21/70 (30%)	24/70 (34.3%)	10/70 (14.3%)	9/70 (12.8%)	8/70 (11.4%)	6/70 (8.6%)
UI episodes/ day *n* (%)	11/70 (15.7%)	12/70 (17.1%)	7/70 (10%)	7/70 (10%)	4/70 (5.7%)	5/70 (7.1%)	4/70 (5.7%)	6/70 (8.6%)	3/70 (4.3%)	5/70 (7.1%)
UF (Q.max) (mean ± SD)	20.1 ± 6.1	24.1 ± 5.2	19.5 ± 5.1	23.3 ± 4.2	21.2 ± 6.2	24.7 ± 5.6	20.1 ± 6.1	22.5 ± 7.1	22.7 ± 5.3	20.1 ± 6.1
PVR (mean ± SD)	20.1 ± 7.2	18.1 ± 8.9	19.8 ± 8.2	18.9 ± 9.1	21.7 ± 7.3	19.1 ± 7.5	18.9 ± 7.5	18.8 ± 9.2	20.8 ± 8.2	18.9 ± 7.3
VAS (mean ± SD)	4.5 ± 0.3	4.7 ± 0.1	5.7 ± 0.8	5.9 ± 0.3	6.2 ± 1	6.5 ± 0.7	6.5 ± 0.8	6.4 ± 0.5	7.3 ± 0.5	7.5 ± 0.3

VAS scores range from 0 (worst) to 10 (best), with higher scores indicating lower pain intensity.

Voiding dysfunction symptoms, reported by 51.4% of patients at baseline, progressively decreased during follow-up, reaching 23.5% at 12 months (*p* > 0.05). Uroflowmetry parameters (Qmax) and PVR volumes remained stable throughout the study period in both groups, without significant longitudinal or between-group differences (*p* > 0.05).

Patient-reported outcomes assessed using the ICIQ-FLUTS questionnaire showed a significant improvement from baseline to the 12 months across all domains (Filling, Voiding, and Incontinence) in both treatment arms. In the Filling domain, mean scores decreased from 6.52 ± 1.28 to 2.33 ± 0.79 in Group A and from 5.89 ± 1.33 to 2.34 ± 1.02 in Group B (both *p* < 0.001 vs. baseline). In the Voiding domain, mean scores decreased from 2.09 ± 0.59 to 1.05 ± 0.38 in Group A and from 2.10 ± 0.63 to 1.15 ± 0.41 in Group B (both *p* < 0.001 vs. baseline). Similarly, in the Incontinence domain, mean scores decreased from 2.59 ± 1.08 to 0.81 ± 0.30 in Group A and from 2.19 ± 0.61 to 0.88 ± 0.31 in Group B (both *p* < 0.001 vs. baseline). No statistically significant differences were observed between Group A and Group B in any of the three domains at baseline or at 12 months, nor in the magnitude of change over time (all *p* > 0.05).

Intravesical administration of HA + CS was well tolerated in both treatment groups throughout the 12-month follow-up period. No serious adverse events related to the instillation procedure or to the study treatment were reported. Mild transient discomfort during catheterization was reported in 3 of 140 patients (2.1%) and resolved spontaneously without the need for medical intervention. No cases of treatment discontinuation due to adverse events were recorded. No episodes of acute urinary retention, macroscopic hematuria requiring intervention, febrile UTI related to the procedure, or systemic reactions were observed. UF parameters and PVR volumes remained stable during follow-up, further supporting the absence of negative functional impact. The safety profile was comparable between the two instillation protocols, with no statistically significant differences in the incidence of adverse events.

## Discussion

This prospective multicentre study demonstrates that intravesical HA + CS is an effective and well-tolerated strategy for the prevention of r-UTIs, leading to a marked reduction in recurrence rates and significant improvement in LUTS over a 12-month follow-up.

Both instillation protocols resulted in a substantial decrease in UTI episodes compared with the pre-treatment period, confirming the clinical relevance of GAG layer restoration as a non-antibiotic preventive strategy. The magnitude of recurrence reduction observed in our cohort is consistent with previous clinical trials and systematic reviews supporting intravesical HA + CS in r-UTI management (6–8). The pathophysiological rationale underlying this therapeutic approach is supported by evidence demonstrating the central role of GAG layer impairment in bacterial adhesion, urothelial inflammation, and increased susceptibility to infection (5). The role of urothelial barrier dysfunction and GAG layer impairment has been widely described in inflammatory bladder conditions, although the relationship between urinary GAG levels and barrier integrity remains complex and not directly linear. Disruption of the GAG layer increases bladder permeability, facilitating both bacterial adherence and exposure of the suburothelial nerve plexus to irritative stimuli (10). This process promotes neurogenic inflammation and sensory hypersensitivity, contributing to symptom persistence and recurrence. By contributing to urothelial barrier restoration, intravesical HA and CS may potentially reduce bacterial adherence and inflammatory urothelial irritation (11). Beyond symptomatic control, these findings support the hypothesis that intravesical GAG replenishment may contribute to symptom control through urothelial barrier support mechanism. Collectively, these findings may support the hypothesis of partially overlapping mechanism across chronic inflammatory bladder conditions, including r-UTIs and chemical cystitis (12). Importantly, no significant differences were detected between the two protocols in terms of UTI recurrence, symptom improvement, or patient-reported outcomes. These findings indicate that no statistically significant differences in clinical outcomes were observed between the 4-week and 8-week induction groups over the 12-month follow-up. The absence of between-group differences should not be interpreted as a lack of treatment effect, but rather as evidence that extending the induction phase beyond four weeks does not confer additional measurable benefit within the observed timeframe. These data contribute to the ongoing discussion regarding the absence of a standardized instillation schedule in current practice (6, 7). Previous evidence has suggested the clinical benefit of intravesical HA + CS administered with an induction phase followed by maintenance instillations. In a large multicentre survey, Cicione et al. reported favourable outcomes using a protocol consisting of weekly instillations for four weeks followed by monthly maintenance, confirming the effectiveness of this regimen in reducing recurrence rates and improving symptoms. However, that study was retrospective in design and did not include a direct comparison between different instillation schedules (13). To date, no prospective studies have directly compared two induction protocols of intravesical HA + CS therapy in women with r-UTIs. To our knowledge, this is the first prospective multicentre study directly comparing two induction schedules on intravesical HA + CS in women with r-UTI within a 12-month follow-up framework. By addressing the current lack of protocol standardization, our findings provide clinically actionable data for daily practice. The absence of a clear superiority of the more intensive induction protocol is clinically relevant. The absence of statistically significant differences between the two treatment groups suggests that further investigation of shorter induction schedules may be warranted, particularly in terms of treatment burden and sustainability. In routine practice, treatment adherence, patient burden, and procedural tolerability are critical determinants of long-term success. A shorter induction phase may improve compliance while maintaining comparable effectiveness, thereby optimizing the balance between therapeutic intensity and sustainability. These findings may help inform future efforts toward protocol standardization in GAG-based therapies.

The improvement in storage and voiding symptoms observed in parallel with the reduction in UTI recurrence further supports the hypothesis that restoration of the urothelial GAG layer contributes not only to infection prevention but also to functional recovery of bladder barrier integrity (5, 7). Importantly, the significant improvement observed in patient-reported outcomes, including all ICIQ-FLUTS domains, underscores the patient-centered impact of this therapeutic approach beyond microbiological endpoints. This dual action is particularly relevant in women with longstanding r-UTIs, in whom chronic inflammation and sensory hypersensitivity may persist even in the absence of active infection (14). By targeting urothelial dysfunction rather than solely eradicating bacteria, HA + CS therapy may contribute to a more comprehensive disease-modifying approach.

Stability of UF parameters and PVR volumes in our cohort further supports the favourable safety profile of intravesical HA + CS therapy. In line with our findings, Damiano et al. demonstrated that intravesical HA and CS significantly reduced UTI recurrence without inducing functional deterioration or worsening of voiding parameters (6).

From a health economics perspective, the absence of superiority of the more intensive induction protocol has clinically meaningful implications. Recurrent urinary tract infections are associated with substantial direct and indirect healthcare costs, including repeated medical consultations, diagnostic testing, antibiotic prescriptions, and productivity loss (2). In this context, therapeutic strategies that maintain clinical effectiveness while reducing procedural burden and resource utilization are particularly valuable. Given the absence of statistically significant differences in efficacy and safety outcomes between the two treatment groups, the shorter induction protocol may warrant further evaluation as a potentially more cost-conscious and sustainable option, decreasing the number of catheterizations, outpatient visits, and overall treatment-related expenditures without compromising clinical benefit. In healthcare system facing increasing economic constraints and rising antimicrobial resistance, optimizing intravesical protocols to minimize procedural burden without compromising effectiveness may represent a rationale and scalable strategy. Furthermore, the economic impact of antimicrobial resistance should not be underestimated. Prolonged or repeated antibiotic prophylaxis contributes to resistance development and increases long-term healthcare costs (15). In the current era of antibiotic stewardship, non-antibiotic preventive approaches supported by prospective data and mechanistic rationale may offer not only clinical but also economic advantages. Preliminary reports have also described the use of intravesical HA + CS in complex cases of r-UTIs caused by multidrug-resistant organisms, suggesting a potential role of GAG replenishment strategies in settings where antibiotic options are limited (16).

These findings support the potential role of structured intravesical GAG replenishment protocols within standardized management pathways for women with r-UTIs, balancing therapeutic effectiveness, sustainability, and healthcare resource optimization. Future cost-effectiveness analyses incorporating quality-adjusted life years (QALYs) and long-term resistance-related expenditures are warranted to further clarify the economic positioning of GAG-based therapies.

Key strengths of the present study include its prospective design, multicentre execution in high-volume referral centres with dedicated expertise in female LUT disorders, balanced treatment groups, and the systematic assessment of both objective and patient-reported outcomes over a 12-month follow-up using validated instruments. The rigorous application of predefined inclusion and exclusion criteria, together with the absence of patient dropout, further reinforces the internal consistency and reliability of the findings. The 12-month follow-up adopted in the present study is comparable to, and in some cases longer than, that reported in most prospective studies on intravesical HA + CS therapy in r-UTIs, where long-term data beyond one year remain limited. Long-term prospective data beyond 12 months remain scarce in the literature, and sustained effectiveness over one year represents a clinically meaningful endpoint in the management of r-UTIs. Beyond their statistical significance, the observed improvements in ICIQ-FLUTS and VAS scores are also likely to be clinically meaningful, as they reflect a reduction in lower urinary tract symptoms and symptom-related burden. These findings suggest an improvement in patients' daily functioning and quality of life, supporting the concept that intravesical HA + CS therapy may provide clinically relevant benefits extending beyond the reduction of recurrent UTI episodes.

The absence of a placebo or untreated control group represents a limitation of the present study and reflects its real-world observational design. However, the primary aim was not to establish treatment efficacy *per se*, but to compare two commonly used instillation protocols in routine clinical practice.

Treatment allocation was based on the predefined routine clinical practice of each participating centre rather than formal randomization. Each participating centre consistently used a single predefined instillation protocol throughout the study period; therefore, all consecutive eligible patients enrolled at that centre received the same treatment regimen. Although this pragmatic design reflects real-world clinical practice, it may have introduced centre-specific selection bias and residual confounding related to local patient populations or management strategies. Baseline characteristics were comparable between groups, and exploratory sensitivity analyses accounting for participating-centre variability did not materially modify the primary outcome. No additional multivariable adjustment was performed, as the primary objective was to compare two standardized treatment protocols adopted in routine clinical practice rather than to identify independent predictors of outcome. Nevertheless, the comparability of measured baseline characteristics does not exclude residual confounding, and the potential influence of unmeasured or incompletely assessed variables cannot be completely excluded, as is inherent to the observational design of the study. In addition, several clinically relevant variables that may influence the risk of recurrent urinary tract infections, including menopausal status, sexual activity, vaginal estrogen use, previous antibiotic prophylaxis duration, microbiological profile, and antimicrobial resistance patterns, were not systematically collected across participating centres because of the pragmatic design of the study. Future prospective randomized controlled trials incorporating these clinically relevant variables, together with predefined cost-effectiveness endpoints, are warranted to further refine the optimal instillation schedule, identify predictors of treatment response, and clarify the role of GAG replenishment therapy within guideline-based management of r-UTIs (1).

## Conclusion

In this prospective multicentre study, intravesical HA + CS were associated with a significant reduction in UTI recurrence and sustained improvement in LUTS and pelvic pain over 12 months. No statistically significant differences in clinical outcomes were observed between the two treatment groups over the 12-month follow-up. Both protocols were well tolerated and did not negatively impact uroflowmetry parameters. These findings further support the role of intravesical GAG replenishment as a non-antibiotic strategy in women with recurrent UTIs.

## Data Availability

The datasets generated and/or analyzed during the current study are not publicly available due to patient confidentiality and privacy considerations. Data may be made available by the corresponding author upon reasonable request, subject to applicable ethical requirements.
